# Differentiation of human umbilical cord mesenchymal stem cells into germ-like cells in mouse seminiferous tubules

**DOI:** 10.3892/mmr.2015.3528

**Published:** 2015-03-23

**Authors:** HUI CHEN, QIU-LING TANG, XIAO-YING WU, LI-CHUN XIE, LI-MIN LIN, GU-YU HO, LIAN MA

**Affiliations:** 1 Department of Neurosurgery, The Second Affiliated Hospital of Shantou University Medical College, Shantou, Guangdong 515041, P.R. China; 2 Department of Pediatrics, The Second Affiliated Hospital of Shantou University Medical College, Shantou, Guangdong 515041, P.R. China; 3 Department of Transformation Medical Center, The Second Affiliated Hospital of Shantou University Medical College, Shantou, Guangdong 515041, P.R. China; 4 Department of Pediatrics, Shenzhen Pingshan Maternal and Child Health Hospital, Shenzhen, Guangdong 518122, P.R. China

**Keywords:** human umbilical cord mesenchymal stem cells, germ cells, *in vivo* differentiation, seminiferous tubules

## Abstract

Our previous study demonstrated that human umbilical cord mesenchymal stem cells (HUMSCs) were capable of differentiation into germ cells *in vitro*. To assess this potential *in vivo*, HUMSCs were microinjected into the lumen of seminiferous tubules of immunocompetent mice, which were treated with busulfan to destroy endogenous spermatogenesis. Bromodeoxyuridine labeling studies demonstrated that HUMSCs survived in the tubule for at least 120 days, exhibited a round cell shape typical of proliferating or differentiating germ cells, migrated to the basement of the tubule, where proliferating spermatogonia reside and returned to the luminal compartment, where differentiating spermatids and spermatozoa reside. The migration pattern resembled that of germ cell development *in vivo*. Immunohistochemical and colocalization studies revealed that transplanted HUMSCs expressed the germ cell markers octamer-binding transcription factor 4, α6 integrin, C-kit and VASA, confirming the germ cell differentiation. In addition, it was observed that tubules transplanted with HUMSCs exhibited marked improvement in the histological features damaged by the chemotherapeutic busulfan, as judged by morphology and quantitative histology. Taken together, these data demonstrated the capacity of HUMSCs to form germ cells in the testes and to repair testicular tissue. These findings suggest a potential utility of HUMSCs to treat the infertility and testicular insufficiency caused by cancer therapeutics.

## Introduction

Infertility affects 15% of couples and half of this is due to reproductive problems in males (1). The pathogenesis of male infertility is due to the inability of germ cells to proliferate and differentiate or somatic-cell dysfunction (2). Several cases of male infertility can be treated by assisted reproductive techniques. However, infertility due to azoospermia or sperm deformity has been a challenge for reproductive medicine (3). In addition, cancer patients may suffer from reproductive failure due to the adverse effect of cancer therapeutics. Stem cell therapy has thus attracted significant interest.

Previous studies have demonstrated that embryonic stem cells (ESCs) (4–11), bone-marrow stem cells (BMSCs) (12), induced pluripotent stem cells (13) and stem cells derived from fetal porcine skin or rat exocrine pancreas can be induced to differentiate toward a germ-cell lineage *in vitro* (14,15). Furthermore, transplanted mouse BMSCs have been demonstrated to form germ cells *in vivo* (16). While studies using ESCs (4–11), BMSCs (12) or other sources of stem cells (13–15) have been instrumental in shedding light onto the potential of germ cell differentiation, their clinical applications may be limited due to ethical challenges or difficulties in obtaining sufficient quantities. Thus, identifying an ideal source of stem cells for use in infertility treatment is being pursued.

Previous studies have indicated that human umbilical cord mesenchymal stem cells (HUMSCs) are a novel source of multipotent stem cells. These cells exhibit a fibroblast-like morphology, express mesenchymal markers, possess pluripotent characteristics for indefinite proliferation and are able to differentiate into advanced derivatives of all three germ layers, including osteocytes, chondrocytes, adipocytes, cardiomyocytes, islet cells and neurons (17–21). Several studies have also demonstrated that HUMSCs express low levels of human leukocyte antigen (HLA)-ABC and do not express HLA-DR (22,23), thus rendering them immunodeficient. In addition, HUMSCs are immunosuppressive in mixed lymphocyte assays (24). The low risk of host rejection coupled with the large donor pool, rapid availability and lack of ethical complications in use, renders HUMSCs a good cell source for use in regenerative medicine.

Our previous study demonstrated that HUMSCs, cultured in a testicular-cell-conditioned medium containing retinoic acid and testosterone, underwent morphological changes and expressed the germ-cell markers octamer-binding transcription factor 4 (Oct-4; POUF5), α6 integrin (CD49f), Stella (DDPA3), C-kit and VASA (DDX4) (25). The present study aimed to investigate the differentiation potential of HUMSCs *in vivo* by transplanting them into the seminiferous tubules of mice treated with the chemotherapeutic busulfan. In addition, the effect of HUMSCs in repairing the structural damage to the testes caused by the cancer drug was examined.

## Materials and methods

### Isolation and expansion of HUMSCs

Isolation and expansion of HUMSCs was performed, as previously described (25) and methods for obtaining the human umbilical cord were approved by the Institutional Review Board of Shantou University Medical College (Shantou, China). Briefly, human umbilical cords were obtained from patients providing written, informed consent and delivering full-term male infants by cesarean section at the Second Affiliated Hospital of Shantou University Medical College. Following removal of the arteries and veins, the remaining tissue, Wharton’s jelly, was transferred to a sterile container in high glucose Dulbecco’s modified Eagle’s medium (H-DMEM; Gibco-BRL, Carlsbad, CA, USA) and diced into small fragments. The explants were transferred into 24-well plates in fresh growth medium (H-DMEM containing 10% fetal bovine serum, 100 mg/ml penicillin, 100 mg/ml streptomycin and 1 mg/ml amphotericin B) and left undisturbed for 5–7 days at 37°C in a humidified incubator with 5% CO_2_ to allow migration of cells from the explants, during which, the media was replaced every 2 days. When the cells reached 80–90% confluence, they were harvested using a 0.05% trypsin/0.53 mM EDTA (Sigma-Aldrich, St. Louis, MO, USA) solution and re-plated into larger culture flasks at a 1:3 ratio.

### Flow cytometry

HUMSCs at passage three were analyzed using flow cytometry to examine the expression of pluripotent cell markers. Following trypsinization, ~1×10^6^ cells were pelleted, resuspended in phosphate-buffered saline (PBS) and fixed with 4% buffered paraformaldehyde (Gibco-BRL) for 20 min at room temperature. Cells were then incubated with monoclonal mouse anti-human antibodies against phycoerythrin (PE)-conjugated CD29 and CD59, or fluorescein isothiocyanate (FITC)-conjugated CD44 (BD Biosciences, Franklin Lakes, NJ, USA). Cells were incubated in the dark for 30 min at 4°C. In order to detect the presence of Oct-4, the cells were permeabilized in PBS with 1% Triton X-100 for 10 min at room temperature, fixed and incubated with a monoclonal mouse anti-human antibody against Oct-4 (Santa Cruz Biotechnology, Inc., Santa Cruz, CA, USA) overnight at 4°C. Cells were then stained in the dark with a PE-conjugated secondary antibody for 30 min at 4°C. Control samples were incubated with FITC or PE-conjugated mouse IgG1 isotype antibodies (Santa Cruz Biotechnology, Inc.). Following incubation, the cells were washed with PBS, centrifuged at 200 × g for 10 min to remove any unbound antibodies, resuspended in 1 ml PBS and analyzed using EPICS XL flow cytometry (Beckman-Coulter Inc., Miami, FL, USA).

### Animal preparation

Male Kunming mice were purchased from the Laboratory of Traditional Chinese Medicine, Guangzhou University (Guangzhou, China). The animals were maintained under standard laboratory conditions (12 h light/12 h dark). Mice at 4 weeks of age received a single intraperitoneal injection of busulfan (Sigma-Aldrich) at 40 mg/kg to destroy endogenous spermatogenesis (26). All animal experimental methods were approved by the Institutional Animal Care and Use Committee of Shantou University Medical College.

### Cell labeling and transplantation

Cultured HUMSCs at passage three were divided into three groups, one stained with Hoechst 33258, a second labeled with bromodeoxyuridine (BrdU) and the third transfected with pIRES2-enhanced green fluorescence protein (EGFP; Clontech Laboratories, Inc., Mountain View, CA, USA). Hoechst staining was performed by incubating cells with Hoechst vital dye (bisbenzimide H 33258; Sigma-Aldrich) in the growth medium at a concentration of 10 ng/ml for 90 min at 37°C. For BrdU labeling, BrdU (Sigma-Aldrich) was added to the log-phase growing cells in the growth medium at a concentration of 5 *μ*g/ml and incubated for 24 h prior to transplantation. Transfection of pIRES2-EGFP into HUMSCs was performed using Lipofectamine™ 2000 (Invitrogen Life Technologies, Carlsbad, CA, USA) according to the manufacturer’s instructions. The transfection efficiency was assessed 48 h later under a fluorescent microscope (DM 2500; Leica Camera AG, Solms, Germany).

Cells labeled using the three methods were washed and resuspended in DMEM. The cell concentration was adjusted to 5×10^6^/ml and ~15 *μ*l cell suspension (2.5×10^5^ cells) was micro-injected into the seminiferous tubules of mice 4 weeks after busulfan administration. The transplantation procedure was conducted using the method previously described (27). Briefly, the mice were anesthetized and placed in dorsal recumbence to expose the testis. The cell suspension was then injected with a microinjector (Karl Storz GmbH & Co, Tuttlingen, Germany) into three to four sites of each testis under a stereomicroscope (Motic China Group, Co., Ltd., Xiamen, China). Busulfan-treated testes without transplantation and testes from normal mice were used as controls. The host mice did not receive any immunosuppressive agent.

### Immunohistochemistry and immunofluorescence

Mice were sacrificed by cervical dislocation at 3, 9, 18 and 20 days, and the testes were dissected, decapsulated and processed, as described below.

To visualize the Hoechst 33258 staining, testes were frozen and cut into 5-*μ*m sections. Frozen slides were shielded from light, fixed in cold acetone for 10 min and air-dried. Testicular sections were then observed under a fluorescent microscope (Olympus BX60; Olympus, Tokyo, Japan) at a wavelength of 346 nm.

For immunohistochemical analysis of the transplanted HUMSCs, the testes were fixed with formaldehyde, embedded in paraffin and cut into 5-*μ*m sections. These were then deparaffinized, rehydrated by successive series of ethanol and rinsed in distilled water. Antigen retrieval was performed in 0.01 mol/l citric buffer (Shanghai Enzyme-linked Biotechnology Co., Ltd, Shanghai, China) at pH 6.0 in a pressure oven, sections were then washed with 0.1 mol/l PBS and incubated in 3% H_2_O_2_ to quench endogenous peroxidase. This was followed by washing with 0.1 mol/l PBS. Sections were blocked with 5% bovine serum albumin for 20 min, incubated overnight at 4°C with the monoclonal primary antibodies of goat anti-human VASA (12 *μ*g/ml; R&D Systems, Minneapolis, MN, USA), mouse anti-human α6 integrin (1:150; Abcam, Cambridge, UK), mouse anti-human Oct-4 (1:60; Santa Cruz Biotechnology, Inc.), mouse anti-human C-kit (1:70; Zhongshan Co., Beijing, China), at the same time and mouse anti-BrdU (1:50; Wuhan Boster Biological Technology, Ltd., Wuhan, China). Following washing to remove unbound primary antibodies, the sections were incubated with monoclonal biotinylated secondary antibodies of rabbit anti-goat IgGs or goat anti-mouse IgGs (1:50; Wuhan Boster Biological Technology, Ltd.). The avidin-biotinylated horseradish peroxidase complex was visualized using 3,3′diaminobenzidine tetrahydrochloride (Wuhan Boster Biological Technology, Ltd.). Slides were counterstained with hematoxylin & eosin (H&E) and viewed under a microscope (DM IRE2, Leica Camer AG). The testes from the busulfan-treated mice without transplantation and from the normal mice were processed identically to the negative controls.

For immunofluorescent analysis of colocalization of EGFP with germ-cell markers expressed by transplanted HUMSCs, testicular sections were processed immunohistochemically, as described above, however, the secondary antibodies used were Cy3-labeled rabbit anti-goat IgGs or Cy3-labeled sheep anti-mouse IgGs (Sigma). Slides were washed and mounted in mounting medium and viewed under a fluorescent confocal microscope (TCS SP5; Leica Camera AG). The testes without transplantation were processed identically to the negative controls.

### Measurement of mean diameter and cross-sectional area of seminiferous tubules

Testicular sections were processed for H&E staining prior to measuring the mean diameter and cross-sectional area of the seminiferous tubules using a high multiple image analytical system (HMIAS-2000; Jinma Medical Instrument, Inc., Xian, China). The system randomly selects 10 areas in a testicular section for measurement, from which mean values are automatically generated. Eight testicular sections from each experimental group were analyzed using this system.

### Statistical analysis

SPSS version 13.0 software (SPSS, Inc., Chicago, IL, USA) was used for statistical analyses. Data were analyzed using a paired t-test. P<0.05 was considered to indicate a statistically significant difference.

## Results

### Cellular characteristics of HUMSCs in primary culture

**Figure 1:**
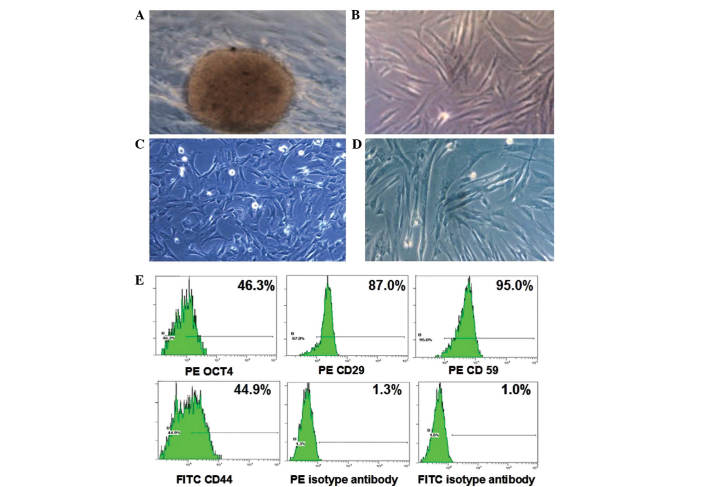
Cellular characteristics of HUMSCs. (A) HUMSCs were observed to migrate out of the tissue fragment and exhibited a fibroblast-like morphology at passages (B) 1, (C) 3 and (D) 9. (E) Flow cytometric analysis of HUMSCs using antibodies against the human stem cell markers octamer-binding transcription factor 4, CD29, CD44 and CD59. PE- or FITC-labeled isotype antibodies were used as controls. Magnification, ×100. HUMSCs, human umbilical cord mesenchymal stem cells; PE, phycoerythrin; FITC, fluorescein isothiocyanate.

A primary culture of HUMSCs was established by growing tissue fragments of Wharton’s jelly from the human umbilical cord in tissue culture plates. Tissues grew adherent to the plate and, after 5–7 days culture, the HUMSCs started to migrate out from the explant (Fig. 1A). The medium was replaced every 2 days until the cells reached 90% confluence prior to passages. At passages one to nine, the cells were fibroblast-like, appeared flat, spindle-shaped or polygonal (Fig. 1B–D). They grew with the doubling time of ~36 h and the growth rate gradually decreased following nine passages. To determine whether HUMSCs had multipotent potential, the cells at passage three were assessed for the expression of markers associated with ESCs and adult stem cells using flow cytometry. As shown in Fig. 1E, 46.3% of these cells were positive for the ESC marker Oct-4 and 87.0, 44.9 and 95.0% of the cells were positive for antigens associated with pluripotent adult stem cells CD29, CD44 and CD59, respectively. These data were consistent with our previous study (25) and those of Wang *et al* (28).

### Preparation of germ-cell deficient recipient mice

**Figure 2:**
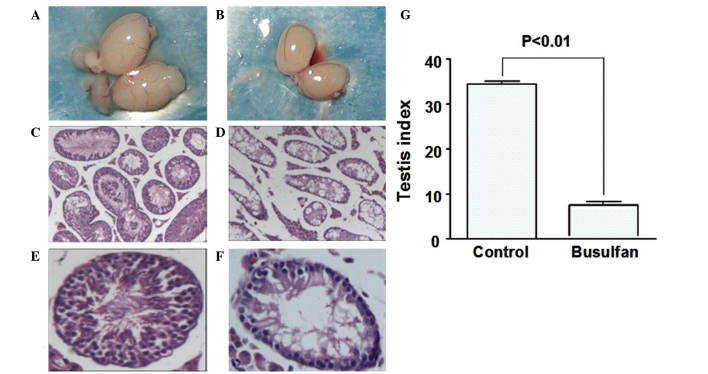
Testes of mice treated with or without busulfan. Mice were treated with or without busulfan and testicular sections were stained with hematoxylin & eosin. Testes of (A, C and E) drug-treated and (B, D and F) untreated mice are shown. (G) The testicular index, defined as testis weight / body weight × 10,000, of treated and untreated mice. Values are expressed as the mean ± standard deviation (eight mice/group). Magnification, (C and D) ×100; (E and F) ×200.

To investigate the germ-cell differentiation of HUMSCs *in vivo*, the recipient mice were administered with 40 mg/kg busulfan to destroy endogenous spermatogenesis (26). Busulfan is the drug of choice to treat myelogenous or granulocytic leukemia, which adversely affects spermatogenesis in mammals (29). It is used in transplantation studies as the treatment provides access for transplanted cells to the basal compartment of tubules and reduces the competition from endogenous germ cells (30). The testes of treated mice appeared smaller than those of untreated testes 4 weeks after the drug treatment (Fig. 2A and B). Quantitative measurement of the mouse testicular index, a ratio of testicular weight / body weight, was 6.5 in the drug-treated group compared with 34.2 in the untreated group (Fig. 2G). Histologically, the seminiferous tubules of treated mice appeared vacuolous (Fig. 2C) with only one layer of germ cells remaining at the periphery (Fig. 2E). By contrast, the tubules of the untreated mice had multiple layers of germ cells, with proliferating spermatogonia at the basement, round spermatids and elongated spermatozoa close to or within the lumen (Fig. 2F). These busulfan-treated mice were then used for subsequent studies.

### Transplantation of HUMSCs into mouse seminiferous tubules

**Figure 3:**
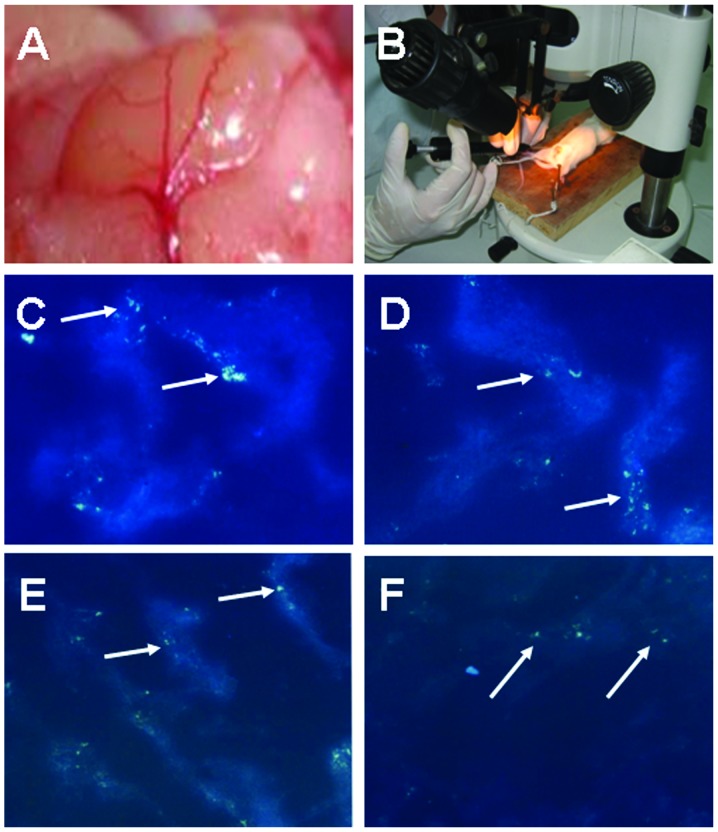
HUMSCs in mouse seminiferous tubules. (A) The testis was visualized under a stereomicroscope to identify the seminiferous tubules for microinjection, following (B) surgery to expose the testis. HUMSCs labeled with Hoechst 33258 (arrows) were examined using fluorescent microscopy (C) 3, (D) 9, (E) 18 and (F) 20 days after transplantation (magnification, ×100). HUMSCs, human umbilical cord mesenchymal stem cells.

In order to assess successful microinjection and initially monitor the survival of HUMSCs in mouse seminiferous tubules, HUMSCs at passage three were stained with Hoechst 33258 and ~2.5×10^5^ cells were injected into the tubule of busulfan-treated mice using a stereomicroscope. The testicular sections were examined at various time points following transplantation by fluorescent microscopy. At days three and nine following transplantation, the HUMSCs were observed within the contour of the seminiferous tubules, distributed predominantly in clusters (Fig. 3C and D). By day 18, the cells appeared dispersed and spread to a wider range within the tubule (Fig. 3E). The fluorescence became weakened with time, nevertheless remained detectable at day 20 (Fig. 3F). The data indicated that microinjection of HUMSCs into seminiferous tubules was amenable and that HUMSCs were able to survive for at least 20 days in immunocompetent mouse testes. The weakened fluorescence may have been due to the fluorescence decay of Hoechst 33258 and the possible proliferation or death of HUMSCs *in vivo*.

### Colonization and differentiation of HUMSCs in mouse seminiferous tubules

**Figure 4:**
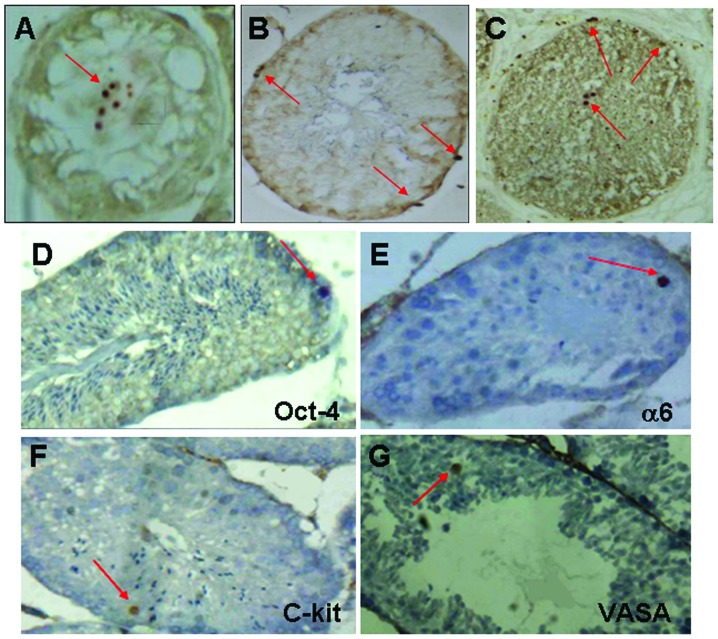
Migration and differentiation of transplanted HUMSCs in mouse seminiferous tubules. Testicular sections transplanted with BrdU-labeled HUMSCs (arrows) were examined by immunohistochemistry (A) 30, (B) 60 and (C) 120 days after transplantation using anti-BrdU antibodies and counterstained with H&E. (D–G) Testicular sections 120 days after transplantation were stained with human-specific antibodies against the germ-cell markers Oct-4, α6 integrin, C-kit and VASA and counterstained with H&E. Testes without HUMSC transplantation were negative for the antibody-staining (data not shown). Magnification, ×200. HUMSCs, human umbilical cord mesenchymal stem cells; BrdU, bromodeoxyuridine; H&E, hematoxylin & eosin; Oct-4, octamer-binding transcription factor 4.

To assess the long-term survival and colonization of HUMSCs in the seminiferous tubules of busulfan-treated mice, HUMSCs were labeled *in vitro* with BrdU prior to transplantation. Colonization of HUMSCs was evaluated at 30, 60 and 120 days after transplantation by immunohistochemistry using anti-BrdU antibodies. At day 30, the majority of the HUMSCs remained in the luminal compartment of the tubules, where the cells were injected (Fig. 4A). At day 60, the majority of cells had migrated to the basement of the tubules (Fig. 4B) where proliferating spermatogonia reside. At day 120, a number of cells had returned to the luminal face where spermatids and spermatozoa reside, while others remained at the basement of the tubules (Fig. 4C). This migration pattern resembled that of germ cell development *in vivo*. During the process, the cells exhibited a round shape (Fig. 4) typical of proliferating/differentiating germ cells. The testes without transplantation were negative for anti-BrdU staining. These data indicated that the HUMSCs survived for at least 120 days in the immunocompetent mouse testes and that the migration pattern and morphology suggested the differentiation of HUMSCs into germ cells.

To confirm the germ cell differentiation, expression of the germ-cell markers Oct-4, α6 integrin, C-kit and VASA by the transplanted HUMSCs was evaluated. α6 integrin is the surface marker of spermatogonial stem cells (31). Oct-4 is enriched in proliferating spermatogonia and downregulated upon differentiation (32–34). VASA is specifically expressed in germ cells from the primordial to the post-meiotic stage (32,35) and C-kit is expressed by early spermatogenic cells, late spermatocytes and spermatids (36,37). In our previous study, these markers were expressed by HUMSCs when induced to differentiate toward the germ-cell lineage *in vitro* (25). Testicular sections were obtained 120 days after transplantation and analyzed using immunohistochemistry with human-specific antibodies against the aforementioned antigens. As shown in Fig. 4D–G, immunopositive cells for these markers were present, which appeared large and round. No immunopositive cells were observed in the testes without HUMSC transplantation. These findings confirmed the germ cell differentiation of HUMSCs in mouse testes.

### Colocalization of EGFP-positive HUMSCs with cells expressing human germ-cell markers in mouse testes

**Figure 5:**
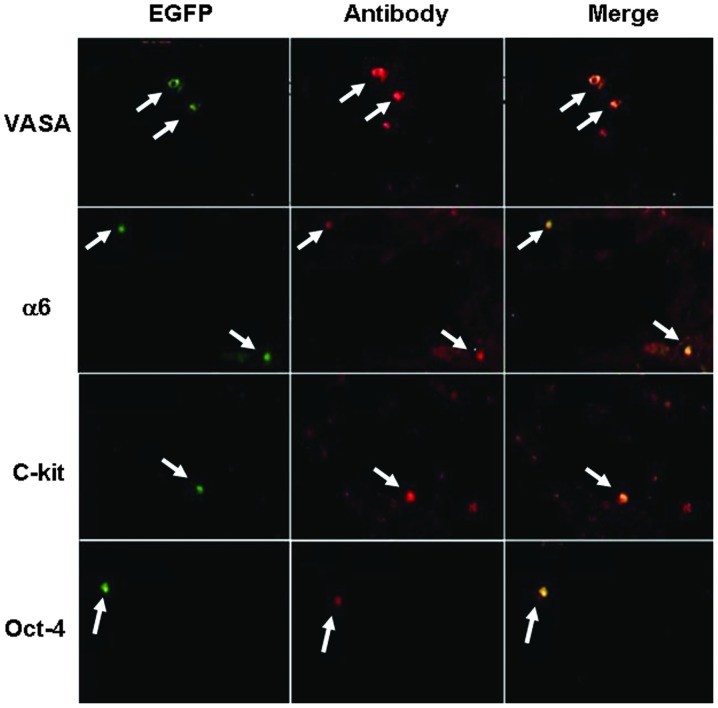
Colocalization of EGFP-positive HUMSCs with antibody-positive cells in mouse testes. Colocalization of EGFP-positive HUMSCs (green) with cells expressing germ-cell markers (red) was assessed by immunofluorescence using human-specific antibodies against the germ-cell markers Oct-4, α6 integrin, C-kit and VASA and visualized by confocal microscopy. Colocalization signals (merge) are shown in yellow. Testes without HUMSC transplantation were negative for the antibody staining (data not shown). Magnification, ×200. HUMSCs, human umbilical cord mesenchymal stem cells; EGFP, enhanced green fluorescence protein; Oct-4, octamer-binding transcription factor 4.

To rule out any cross-reactivity of the antibodies with mouse antigens, HUMSCs were transiently transfected with EGFP-expressing vectors. The transfection efficiency was ~40% and cells were microinjected 48 h post transfection into mouse seminiferous tubules. Colocalization of EGFP-positive cells with cells expressing the germ-cell markers Oct-4, α6 integrin, C-kit and VASA was assessed by immunofluorescent microscopy using the antibodies indicated in Fig. 4D–G. As shown in Fig. 5, the EGFP-positive cells were colocalized with the antibody-positive cells (Fig. 5, Merge). The EGFP signal became weakened with time, however, a similar colocalization result was observed 120 days after transplantation (data not shown). Notably, there were significantly more C-kit-positive cells than EGFP-positive cells in the corresponding field (Fig. 5), implying the possible proliferation of HUMSCs and an upregulation of C-kit. In addition, there were very few Oct-4-positive cells (Fig. 5), which may suggest a downregulation of Oct-4.

### Histological features of mouse seminiferous tubules transplanted with HUMSCs

**Figure 6:**
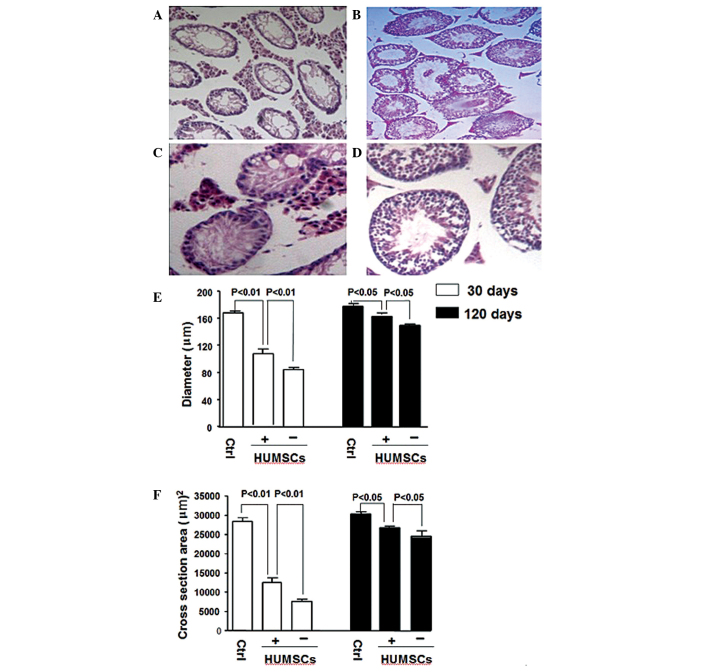
Histology of mouse seminiferous tubules. Testes of busulfan-treated mice with HUMSC transplantation at days (B) 30 and (D) 120 or without transplantation at days (A) 30 and (C) 120 were stained with hematoxylin & eosin. (E) The mean diameter and (F) cross-sectional area of tubules from age-matched control and busulfan-treated mice with or without transplantation were quantified using a high multiple image analytical system. Values are expressed as the mean ± standard deviation (n=8). (A and B) magnification, ×100; (C and D) magnification, ×200. HUMSCs, human umbilical cord mesenchymal stem cell; Ctrl, control.

During the present study, an improvement of testicular histological features following HUMSC transplantation was observed. At day 30 after transplantation, the tubules of busulfan-treated mice had fewer vacuoles and more cells (Fig. 6B) than those of the untransplanted tubules (Fig. 6A). At day 120, the morphological improvement was more pronounced, where transplanted tubules had markedly more germ cells forming a multi-layered cell organization (Fig. 6D), while the untransplanted tubules maintained a mono-layered cell structure (Fig. 6C). The mean diameter and cross-sectional area of the tubules were further analyzed quantitatively using a high multiple image analytical system. At 30 days after transplantation, the mean tubule diameter was 114 *μ*m in the transplantation group compared with 85 *μ*m in the untransplanted group (Fig. 6E), representing a 34% increase; the mean cross-sectional area of tubules was 13,582 *μ*m^2^ in the transplanted group compared with 8,624 *μ*m^2^ in the untransplanted group (Fig. 6F), representing a 58% improvement. The values between the two groups were statistically different and were smaller than the age-matched control mice that did not receive busulfan (mean diameter 168 *μ*m and mean cross-sectional area 27,026 *μ*m^2^). At day 120, the two groups demonstrated further improvement in tubule diameter and cross-sectional area, approaching those of the age-matched control mice (Fig. 6E and F). However, these parameters remained improved in the transplantation group (Fig. 6E and F). These data indicated that transplantation of HUMSCs facilitated the recovery of the tubule morphology damaged by busulfan treatment.

## Discussion

In the present study, the potential of multipotent HUMSCs to differentiate into germ cells was examined by transplanting them into the seminiferous tubules of mice treated with busulfan. The results revealed that HUMSCs survived in the mouse testis for at least 120 days, exhibited a round cell shape typical of proliferating/differentiating germ cells, migrated in a pattern resembling that of germ cell development *in vivo* and expressed the germ-cell markers Oct-4, α6 integrin, C-kit and VASA. The present study also demonstrated that the transplantation of HUMSCs facilitated the structural recovery of the seminiferous tubule damaged by busulfan, as judged by morphology and quantitative histology.

During spermatogenesis in seminiferous tubules, germ cells exist in three main developmental phases: mitosis, meiosis and spermiogenesis. The three phases of germ cells are arranged in a highly organized architecture with spermatogonial stem cells/proliferating spermatogonia present at the basal lamina of the tubule and the post-meiotic spermatids and mature spermatozoa present close to or in the lumen (30). Proliferating or differentiating germ cells exhibit a round cell shape, with the exception of the characteristic appearance of mature spermatozoa (30). Using BrdU labeling to follow the survival and colonization of transplanted HUMSCs in mouse seminifierous tubules, the present study demonstrated that transplanted HUMSCs migrated to the basal compartment, which was followed by returning back towards the luminal compartment. During the migration process, these cells were observed as single round cells. The morphology and migration pattern of transplanted HUMSCs are thus consistent with germ cell development *in vivo*.

Molecular markers are specifically expressed or enriched during each stage of germ cell development. Immunohistochemical and colocalization studies revealed that transplanted HUMSCs expressed the germ-cell markers Oct-4, α6 integrin, C-kit and VASA 30 days after transplantation and the expression remained at least 120 days after transplantation. In the present study, Oct-4 was also found to be expressed by undifferentiated HUMSCs. However, the expression of α6 integrin, C-kit and VASA occurred only in the transplanted HUMSCs, thus confirming germ-cell differentiation. Of these molecular markers, Oct-4 is expressed by proliferating spermatogonia and downregulated upon differentiation (32–34). C-kit has been demonstrated to be expressed by all stages of germ cells (37), although a previous study indicated that it is expressed only by late spermatocytes and round spermatids (36). The observation that there may be a downregulation of Oct-4 with a concomitant upregulation of C-kit in HUMSC-derived germ cells may suggest that these cells may have differentiated into late stages of germ cells. Further studies to examine the expression of meiotic markers, including SCP1 and SCP3 are required to confirm the progression through the meiotic phase.

The present study demonstrated that HUMSCs survived in immunocompetent mouse testes for at least 120 days. HUMSCs have been demonstrated to have low immunogenicity (22,23), as well as to possess immunosuppressive activities (24). Transplanting HUMSCs into the brain of immunocompetent rats did not provoke a host immune rejection in the xenogeneic background, with transplanted cells surviving for at least 4 months (23). The present study provides additional support for the low-risk of host rejection and the potential long-term survival of HUMSCs in the allogeneic background of clinical applications.

Busulfan is the drug of choice to treat myelogenous or granulocytic leukemia, which adversely affects spermatogenesis in mammals (29). High doses of busulfan (40 mg/kg) eradicates germ cells, sterilizes mice and results in long-term morphological damage to sperm produced by the surviving spermatogonial stem cells (26). However, other studies have demonstrated that spermatogenesis is able to partly recover following two spermatic cycles (38). Notably, the present study demonstrated a marked improvement in the histological features of the tubules transplanted with HUMSCs, despite the relatively small fraction of cells (2.5×10^5^) injected into each testis. This observation is of particular significance in the context of the tubule damage caused by the gonadotoxic effect of busulfan and may have far-reaching implications on HUMSCs in the treatment of testicular insufficiency caused by cancer therapeutics.

HUMSCs have been demonstrated to have therapeutic effects in pre-clinical animal models for neurodegenerative disease (23), cancer (39,40) and corneal disease (41). The underlying mechanisms are postulated to be due to the release of trophic factors and dampening of the host immune response to limit secondary inflammatory damage. In the present study, no apparent inflammatory infiltrates in the testes of busulfan-treated mice nor mice transplanted with HUMSCs were observed. It is possible that the trophic factors released by the HUMSCs may be one of the underlying molecular mechanisms in testicular tissue repair. It is also possible that HUMSC-derived germ cells and/or HUMSC-derived Sortoli cells, which were not assessed in the present study but warrant further investigation, may secrete trophic factors, including bone morphogenetic protein 4, leukemia inhibitory factor, stem cell factor and growth differentiation factor-9 that are required for the development of germ cells in the testes (42–45). Furthermore, mechanistic studies, as well as assessment of mouse fertility, are required to extend the initial observation of HUMSCs in testicular tissue repair.

In conclusion, the present study demonstrated that HUMSCs transplanted into the testes of immnuocompetent mice survived long-term, differentiated into germ cells and facilitated the restoration of tubule morphology that was damaged by the chemotherapeutic busulfan. These data demonstrated the capacity of HUMSCs to form germ cells in the testes and to repair the testicular tissue. These findings, together with the low-risk of host rejection, abundant source and lack of ethical complications in use, suggest the potential utility of HUMSCs to treat infertility and testicular insufficiency caused by cancer therapeutics.
